# Editorial

**Published:** 1851-12

**Authors:** 


### THE MEDICAL EXAMINER.

PHILADELPHIA, DECEMBER, 1851.

### NEW ORLEANS MONTHLY MEDICAL REGISTER.

We have the *second* number of this new candidate for professional support—the *first* number having doubtless failed to reach us through the delinquency of the post-office. It is a small but spirited sheet, of the '*Gazette*' order, and presents in the number before us, an interesting series of original and selected papers. The editor, A. FORSTER AXSON, M. D., has our best wishes for his success.

DEATH OF DR. J. M. WALLACE, OF PHILADELPHIA.

The announcement of the death of this gentleman, we doubt not, will reach his distant friends with the same stunning force that it did those in his own city, whom his brief and sudden illness but little prepared for so sad a termination of his most useful life. He was attacked with a severe pleuro-pneumonia on Friday, Nov. 6th., which resisted all treatment, and ended in rapid effusion, under which he sank on Monday, Nov. 10th, at 4½ P. M., in the 37th year of his age.

There was perhaps no man in the profession more universally beloved than Dr. Wallace. Young and old, rich and poor, united in the same expression of unmingled regret at his untimely fate. His manliness of character, his modest self-reliance, and his promptitude and decision of action, had secured to him the confidence of men far beyond him in age and professional standing; while his more intimate friends and associates ever found in him a sincerity and truthfulness of disposition and soundness of judgment, that could always be relied upon. He occupied a place in their affections that can never be filled.

The character of his mind was essentially practical, and always fertile in expedients, a trait whose value every surgeon will recognize. As a teacher he was clear and explicit, possessing a remarkable facility in imparting knowledge and impressing it upon his hearers. The high position which he held in all his relations both public and private, will be better estimated by the following resolutions passed by the College of Physicians and by his private class, than by any feeble expression of ours.

—

At a special meeting of the College of Physicians of Philadelphia, held Nov. 12th, 1851, the following preamble and resolutions were unanimously adopted:

Whereas, by the sudden decease of Dr. Joshua M. Wallace, we have been called upon to mourn the loss of one of our most esteemed and promising colleagues, we feel it due to ourselves, to the memory of the deceased, and to the profession of which he gave so fair a promise of becoming one of the brightest ornaments, had not his career of usefulness been so unexpectedly cut short, to give an expression to our sentiments in reference to this melancholy event. Therefore,

*Resolved,* That in the death of Dr. Joshua M. Wallace, we mourn the loss of a colleague endeared to us by the dignity of his moral deportment, the suavity of his manners, and by his professional attainments and skill.

*Resolved,* That we most sincerely condole with the family and relations of the deceased, and tender to them our warmest sympathies in their severe bereavement.

*Resolved,* That Dr. Francis G. Smith be requested to prepare a biographical notice of the deceased, to be read before the College.

*Reeolved,* That Drs. John Neill, Francis West, and John B. Biddle, be constituted a committee to present, on behalf of the College, a copy of the foregoing preamble and resolutions to the family of the deceased.

D. FRANCIS CONDIE, Sec'y.

---

At a meeting of the private class of the late Joshua M. Wallace, M. D., assembled to express their regret and sorrow for the death of that distinguished and useful gentleman, distinguished both in the capacity of a private citizen, and in that of the profession to which he belonged and which he so much adorned, the following gentlemen were appointed a committee to draft resolutions expressive of their feelings on the occasion:

Charles Neff, Chairman; L. H. Smith, Secretary; E. O. Dummer, M. G. Lofland, James F. Mabrey, A. B. Longman, W. R. Cunningham.

The following report was presented and unanimously adopted:

1st. *Resolved,* That whereas, by the inscrutable decree of Providence, a useful and honorable member of society, as well as of the profession to which we aspire, has been cut down in the midst of his career, and in the prime of life, that we, his private class, though associated with him for but a comparatively short time, had yet sufficiently learned to estimate his qualities, both of mind and heart, as not only to admire, but love him.

2d. *Resolved,* That we feel deeply the loss of one whose instructions we had but just sufficiently experienced to learn their value, and by the aid of which we had indulged the hope that we should be able to accomplish the arduous task before us.

3d. *Resolved,* That with his bereaved family and relations, as well as the numerous friends which one so gifted most deservedly had, we most deeply and truly condole and sympathise.

4th. *Resolved,* That we wear the usual badge of mourning for thirty days after the adoption of these resolutions.

5th. *Resolved,* That a copy of these resolutions be sent to the widow of the late J. M. Wallace, M. D., and to his brother, Ellerslie Wallace, M. D., our much esteemed Demonstrator of Anatomy and private instructor.

6th. *Resolved,* That the foregoing be published in the *Medical News* and *Medical Examiner* of this city.

*Philadelphia,* Nov. 13th, 1851.

---

We have also to record the death of another much respected practitioner of Philadelphia, STEPHEN HARRIS, M. D., which took place after a protracted illness. Dr. Harris was a younger brother of Dr. Thomas Harris of the United States Navy, and also of Dr. William Harris of this city. He had passed most of his life in Chester county, in this State, where he was long engaged in an extensive practice. Within two years past he removed to Philadelphia, and had the best prospects of professional success here, when stricken down by disease. He enjoyed in a high degree the regard and esteem of all those who knew him.

---

Drs. GRANVILLE SHARPE PATTISON, J. KEARNEY ROGERS, and J. R. MANLY, distinguished and well-known physicians of New York, have been summoned hence, within the past month—we believe, quite unexpectedly. Dr. Pattison's death, was, we learn, the result of ulceration of the ductus communis choledochus. Dr. Rodgers also fell a victim to biliary disease—inflammation of the vena portarum. Dr. Manly was advanced in years. Many of our readers may remember him as a delegate to the meeting of the National Medical Association in Philadelphia, where his enthusiastic and able support of the cause of *medical reform* was quite conspicuous.

---

DIED, on the 13th of November, 1851, Doctor FREDERICK CROWLEY, aged 38 years.

At a special meeting of the Northern Medical Association of Philadelphia, of which Dr. Crowley was a member, the following resolution was unanimously adopted.

*Resolved,* That while we deeply deplore the loss of one of our associates, cut off from among us in the prime of his manhood, and in the enjoyment of the active duties of his profession, we feel that the profession at large, of which he was a highly respected member, has sustained a loss in one whose medical attainments and whose social and moral worth were appreciated wherever he was known.

---

Dr. JOHN BASKIN, an eminent practitioner of Mercer county, in this State, died at his residence there, on the 26th of September last, from the effects of injuries received in being thrown from his carriage the preceding day.

The subjoined "slip" from the Charleston Medical Journal and Review, for November last, has been forwarded to us by Dr. F. M. Robertson, of Charleston. We give it place—though certainly a considerable draft upon our space—from a wish to allow a full hearing to all the parties concerned in this controversy. We forbear all further comment on the subject, and with this publication we dismiss it from our columns, which are inappropriate to the discussion of any "collateral issues" developed in the controversy.

[The subjoined "Card," being considered by Dr. F. M. Robertson as a final settlement of the difficulty between Dr. H. A. Ramsay and himself, we insert it, together with his letter to Dr. DeSaussure, at his request. —*Eds. Charleston Medical Journal.*]

A Card.—Since the publication of my reply to the pamphet recently issued by Dr. F. M. Robertson, of Charleston, S. C., I have been in correspondence with Dr. J. J. Robertson, of Washington, Ga., through a friend, and having received from him the grounds upon which the opinion of himself and Dr. D. M. Andrews, in regard to my professional statistics, was based—which opinion led them when called on by members of the American Medical Association, at their late Convention in Charleston, to express a want of confidence in the reliability of my obstetrical statistics, and induced Dr. F. M. Robertson, a member of that Association, to move to strike out the notice of said statistics from the report of Dr. Storer, of Boston—and having received from Drs. Andrews and Robertson the assurance that those opinions were neither formed or expressed through personal ill-will towards myself, but were honestly entertained and given in pursuance of what they regarded their duty to the profession ; and feeling convinced from the representations of these gentlemen that they were influenced by circumstances, which, unexplained, were calculated to impair their confidence in me as reporter, I feel bound in justice to them, to state that, with such such impressions on their minds, they could not, when called on, have given other that an unfavorable opinion, and that Dr. F. M. Robertson, in making the motion he did, under the circumstances, was actuated by no unworthy or improper motive.

The grounds on which Drs. Andrews and J. J. Robertson were led to the statement that I had not, during my practice of physic, been continuous in one place, were my removal from Bookersville to Mrs. Wellborn's, a distance of six miles, and subsequently to this place, a distance of one and a half miles, while I regarded myself as never having left the *neighborhood* in which I originally settled. I have also been satisfied, from the statements and evidence furnished me by Dr. J. J. Robertson, that although he has not had a general practice in my neighborhood, he has made over twenty professional visits in the vicinity of my location within the past six years, a fact of which I had not been apprised at the time my late pamphlet was published.

In issuing this card to the profession and the public, I am impelled by no other motive than a desire to render strict justice to all the parties concerned, and therefore feel bound, under the circumstances, as an honorable man, to withdraw all offensive language I may have used in my pamphlet, or elsewhere, touching this controversy, either towards Drs. Robertson and Andrews, or towards Dr. F. M. Robertson, of Charleston.

I respectfully submit this statement to the profession and the public, with the single additional remark, that it is regarded by all the parties as an amicable and honorable termination of this entire controversy.

*Raysville, Ga., Oct. 14th,* 1851 H. A. Ramsay.

—

Charleston, S. C., Oct. 31st, 1851.

*Dear Doctor:*—Since you handed me the two letters of Dr. H. A. Ramsay to yourself—one dated Sept. 1st, the other Oct. 1st, I have received "a card"—issued by the same gentleman—dated Raysville, Ga., Oct. 14th, 1851, one of which I presume has been sent to you also.

As circumstances growing out of Dr. Ramsay's published defence of August last, and his private correspondence with numerous persons, have drawn me into personal issues with certain parties, which are yet unadjusted, I deem it but just to Dr. Ramsay, as well as due to my own position, to make the following remarks, which will prevent misunderstanding in future.

1st. The card alluded to, was unsolicited by me, but voluntarily tendered by Dr. Ramsay. Nor was the disclaimer, of its author, of imputing "*unworthy or improper motives*" to me, for the course which I thought it my duty to pursue in relation to his obstetrical statistics; or the withdrawal of "*all offensive language in his pamphlet or elsewhere,*" in regard to myself, the result of any correspondence or explanation on my part, either *directly* or *indirectly*.

2. Desiring still, as stated in my note to you, of the 28th of June, published in my circular of July last, to "throw no impediment in the way of his doing himself ample justice in the matter complained of," I now accept the card as an honorable amend for the imputations against my motives, and his personal abuse, and consider it, so far as I am concerned, a final termination of the personal difficulty between Dr. Ramsay and myself.

3d. In as much as the collateral issues in which I have been involved, (by no act of my own however,) with the editors of certain medical periodicals, in relation to the merits of the "Obstetrical Statistics," are yet unsettled, I hold myself perfectly free, until they are satisfactorily arranged—in case it becomes necessary to my defence—to discuss the professional merits of the points of difference, without its being considered a violation of my acceptance of Dr. Ramsay's card.

4th. If the question should be introduced into the American Medical Association, at its next meeting, as has been intimated from certain quarters, I reserve to myself the privilege claimed in the preceding paragraph.

Having thus set forth my position, that it may be clearly understood by all parties, I remain very truly and sincerely,

Your obedient servant,

Dr. H. W. DeSaussure, F. M. Robertson.
Sect. A. M. A., Charleston, S. C.

